# Supplementary material for: Non-viral in vivo electroporation-based chromosomal engineering and repair assessment in the murine uterine epithelium
Source: PLoS One. 2026 May 11;21(5):e0348797. doi: 10.1371/journal.pone.0348797 (PMC13160296; doi:10.1371/journal.pone.0348797)
Supplement: S1 Fig — (PDF) [file pone.0348797.s001.pdf]

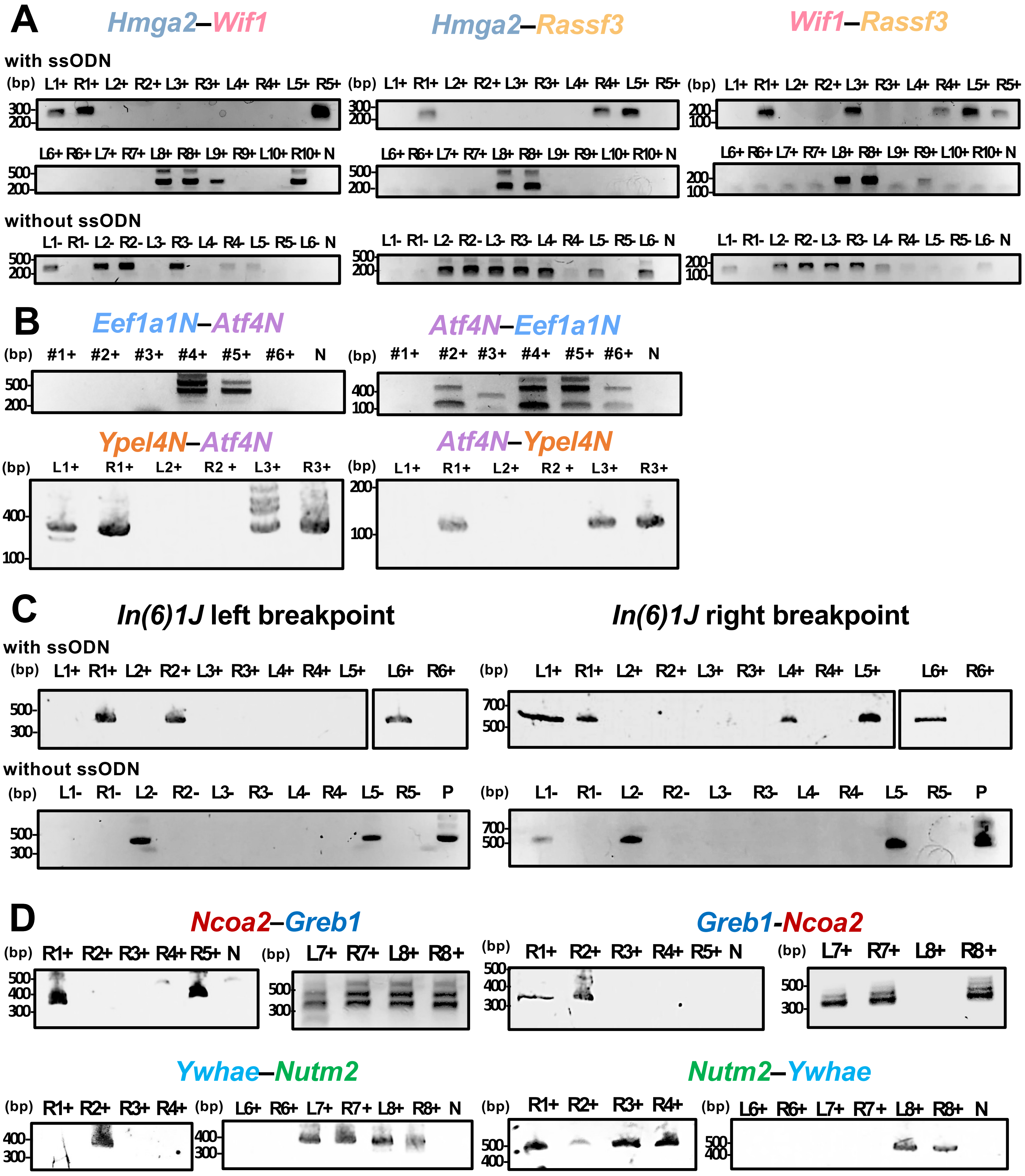

S1 Fig.

Representative PCR genotyping of genomic DNA extracted from uterine horns edited by *in vivo* electroporation.

(A) Complex chromosomal rearrangements (CCRs) among *Hmga2*, *Wif1*, and *Rassf3*.

(B) Translocations involving *Eef1a1N–Atf4N* and *Ypel4N–Atf4N*.

(C) Repair of the large-scale inversion *In(6)1J*.

(D) Translocations involving *Ncoa2–Greb1* and *Ywhae–Nutm2*.

Lanes marked “+” and “–” indicate samples electroporated with or without ssODN donors, respectively. N and P denote negative and positive controls, respectively.
