# Supplementary material for: Non-viral in vivo electroporation-based chromosomal engineering and repair assessment in the murine uterine epithelium
Source: PLoS One. 2026 May 11;21(5):e0348797. doi: 10.1371/journal.pone.0348797 (PMC13160296; doi:10.1371/journal.pone.0348797)
Supplement: S2 Fig — (PDF) [file pone.0348797.s002.pdf]

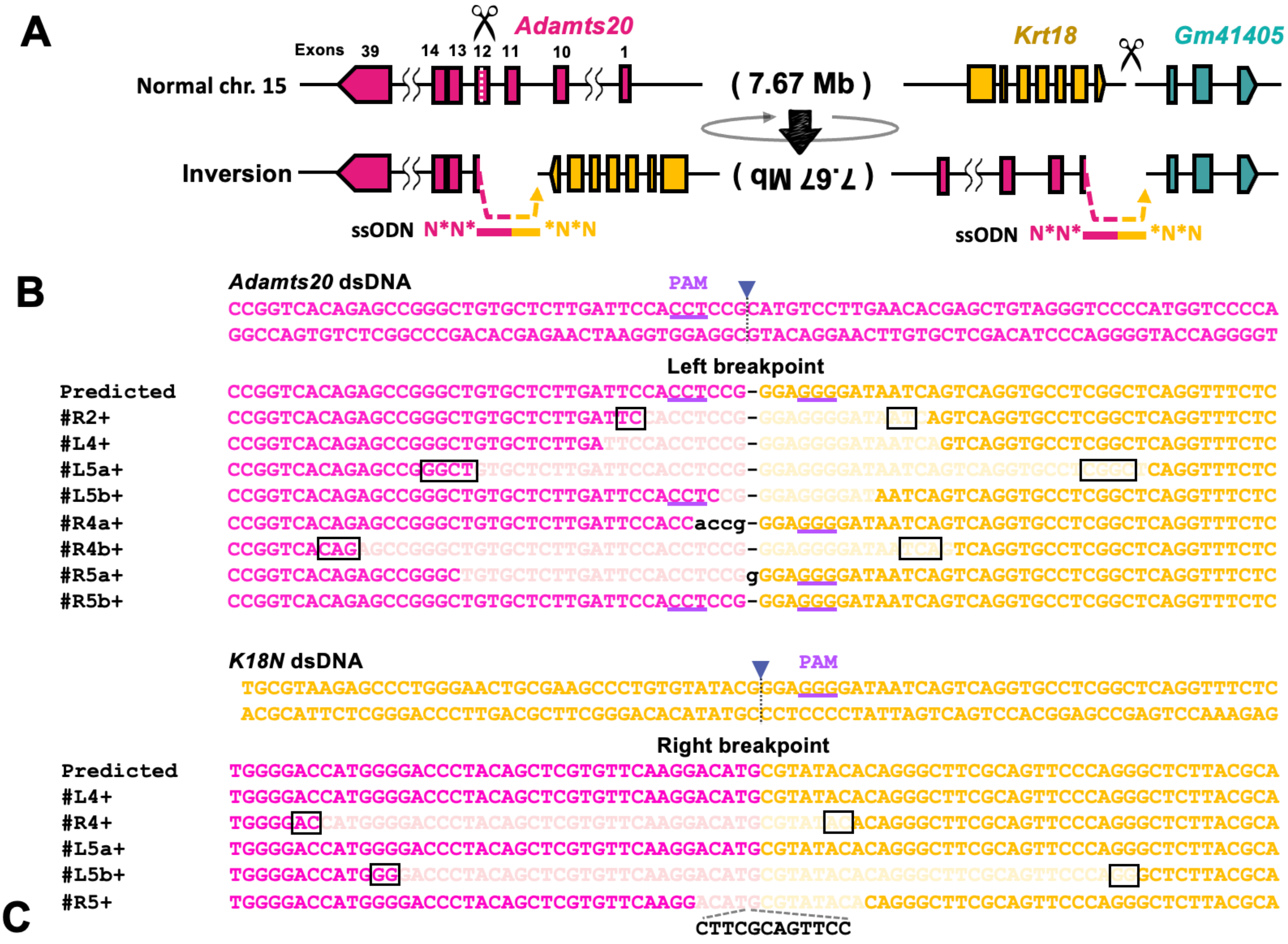

C

Cas9 targets	Distance (Mb)	Uterine horns treated (*n*)	Precise junctions / rearrangements (%)	
			Left breakpoints	Right breakpoints
Adamts20<br>*K18N*	7.67	10	1 / 5 (20)	1 / 4 (25)

S2 Fig.

Inversion in the uterine epithelium induced via *in vivo* electroporation.

(A) Schematic of the 7.67-Mb chromosomal inversion between *Adamts20* and the *Krt18* neighborhood locus (*K18N*) on chromosome 15, showing Cas9 cut sites (scissors) and ssODN donors for junction repair. (B) Sequence alignment of the PCR products corresponding to the genomic breakpoint junctions of *Adamts20* and *K18N* locus. Arrows indicate Cas9 cleavage sites; boxed bases, microhomology; lowercase letters, insertions or mismatches; underlines, PAM sequences. PAM, protospacer adjacent motif; dsDNA, double-stranded DNA. (C) Inversion efficiency of *Adamts20*–*K18N* in the uterine epithelium. Precise junctions denote ssODN-consistent joins; rearrangements, all detected junctions connecting the intended loci.
