# Supplementary material for: Non-viral in vivo electroporation-based chromosomal engineering and repair assessment in the murine uterine epithelium
Source: PLoS One. 2026 May 11;21(5):e0348797. doi: 10.1371/journal.pone.0348797 (PMC13160296; doi:10.1371/journal.pone.0348797)
Supplement: S1 Table — (PDF) [file pone.0348797.s003.pdf]

S1 Table. gRNAs used in the present study.

Target loci	Genomic location (mm10/GRCm38)	Target Sequences (PAM)	CHOPCHOP (https://chopchop.cbu.uib.no/) Number of mismatches 0	1	2	3	Efficiency
Hmga2	chr10: 120416213	GGAGTGTTAGGTCGACCCAA (TGG)	0	0	0	0	67.29
Wif1	chr10: 121049539	ATAGAGCATGCCCAATGGCG (GGG)	0	0	0	0	60.84
Rassf3	chr10: 121431836	ACAGGTACAAGACCCGTCAC (TGG)	0	0	0	2	59.01
Eef1a1N	chr9: 78477382	ACTTATACACGTAAGTGTGC (AGG)	0	0	0	2	55.34
Atf4N	chr15: 80258539	CGTAACAAGGGTGAGCCCAA (AGG)	0	0	0	2	64.88
Ypel4N	chr2: 84738819	CCAGGTGAACCAAGAGGCGT (GGG)	0	0	0	5	59.00
In(6)1J left breakpoint	chr6: 63000847	TGCtcATACCAACTGATATG (AGG)	-				
In(6)1J right breakpoint	chr6: 120827195	TAGTAGTACATCTGGAGGCC (TGG)	-				
Ywhae	chr11: 75760846	GACCAAGCAAGTTCTCACAT (AGG)	0	0	0	5	70.57
Nutm2	chr13: 50467977	AGACATCAACCACCCGATAA (AGG)	0	0	0	3	51.94
Ncoa2	chr1: 13161287	TTATTTGTAACCGTAGACAT (AGG)	0	0	0	7	65.93
Greb1	chr12: 16734411	GTTAGCCAGGAGACTTCCAT (GGG)	0	0	0	5	62.57
Adamts20	chr15: 94347708	TCGTGTTCAAGGACATGCGG (AGG)	0	0	1	3	71.44
K18N	chr15: 102034830	GAAGCCCTGTGTATACGGGA (GGG)	0	0	1	4	61.49
