# Supplementary material for: Non-viral in vivo electroporation-based chromosomal engineering and repair assessment in the murine uterine epithelium
Source: PLoS One. 2026 May 11;21(5):e0348797. doi: 10.1371/journal.pone.0348797 (PMC13160296; doi:10.1371/journal.pone.0348797)
Supplement: S2 Table — (PDF) [file pone.0348797.s004.pdf]

S2 Table. Single-stranded oligodeoxynucleotides (ssODNs) used in the present study.

Target loci	Target Sequences (5′–3′)
Hmga2–*Wif1* (Left)	PAM<br>C*G*TTGCAGCATGTAGGTGTGGTAGCATCTGCACCCACCAAAATACCATTG<br>CATTGGGCATGCTCTATTGAAGGCTGAGTCCAGAGCTCTCCATTGGGC*A*T
Hmga2–*Rassf3* (Middle)	G*A*AGCACGAAGAAAAGAGTTTAAAAATTCAAAGGGAGTGTTAGGTCGACC<br>CACTGGTTAGAAATGTCGGGATTGGTTGCCCCCTGCCTCCTAGGCACT*G*G
Wif1–*Rassf3* (Right)	T*A*ACCCAAAAAAGATTTTTTTAAAAAACCACGCCTTACTGAGAGCCCCGC<br>ACGGGTCTTGTACCTGTAGCCCCATGGGCCATGGGTTCTTGGCTAATT*T*T
Eef1a1N–*Atf4N* t(9:15)	T*G*TCTTCAAAGCACCAGTAGATGGTGCTACTCCACTTATACACGTAAGTG<br>GGCTCACCCTTGTTACGCACAGAAGCTAGGCTGTAAGTAGTTAAGTCT*C*T
Atf4N–*Eef1a1N* t(15:9)	G*G*TGTGGGTAGGATGATACAGCAGCCTCCCACTTCTGCAGCAGGCCTTTG<br>TGCAGGTTTGAAAAACGGTGTTTGTATCCAGCAGACACTTGCTATCAA*T*T
Ypel4N–*Atf4N* t(2:15)	T*C*CTCCTCCACCTGTTTCACAAGCGGTAAGCATCCAGGTGAACCAAGAGG<br>GGCTCACCCTTGTTACGCACAGAAGCTAGGCTGTAAGTAGTTAAGTCT*C*T
Atf4N–*Ypel4N* t(15:2)	G*G*TGTGGGTAGGATGATACAGCAGCCTCCCACTTCTGCAGCAGGCCTTTG<br>CGTGGGTTTTGTGAGCTTGTCCAAAGGGAAGGAATATCTAAGTGTCTT*C*A
In(6)1J left breakpoint	C*T*GGAAGTCCTTTAATATATGTGTGTTGGAGGCCTCATATCAGTTGGTAT<br>ATGCTGCCTGGTTGGTGGTCCAGCGCCTGAGAGATCTTGGGGTTCCAGGTTA<br>A*T*T
In(6)1J right breakpoint	C*T*GGGAACTGACAGGTCCTCGAGAAGAAGAAGAAGGCTCTTAACAACTGC<br>AGCCTCCAGATGTACTACTATGATAATCCAATCCTTAGTAGAGAATAACAG*<br>G*A
Ncoa2–*Greb1* t(1:12)	A*A*GATCAGTTGCCTATACAGCTCCTACTTGATATTATTTGTAACCGTAGA<br>CATGGGTGGGCAAGCTTGGAAACTGCAGAGGAAGCAGTGGCACAAAGG*C*T
Greb1–*Ncoa2* t(12:1)	G*T*CCTTCCTCTGGGTTTTAGATGATCTGTAAGAGTTAGCCAGGAGACTTC<br>CATAGGACTTAAATCTTAAACATTAGCTCAATTCTAAAAAGTCTGTGT*G*T
Ywhae–*Nutm2* t(11:13)	T*T*TCTGGCAAAATTGAGTTTGATTTTTTACTTTATTTTTCCTAACCTATG<br>TCGGGTGGTTGATGTCTTTGATTCTACAGAGGCAGAACAGTTAGGGGG*A*G
Nutm2–*Ywhae* t(13:11)	A*C*TGGCAGTCCAGAGTTCCACCTTCCCACTCAGGGAAGGCTTCTCCTTTA<br>TGAGAACTTGCTTGGTCAGCTTCTGAAAGCTCACTAGGTCATCAAGTA*T*T
Adamts20–*K18N* (Left)	C*G*TACTCGGGCCGGTCACAGAGCCGGGCTGTGCTCTTGATTCCACCTCCG<br>GGAGGGGATAATCAGTCAGGTGCCTCGGCTCAGGTTTCTCGGGTCAGC*T*T
Adamts20–*K18N* (Right)	G*A*TGGCGAGTGGGGACCATGGGGACCCTACAGCTCGTGTTCAAGGACATG<br>CGTATACACAGGGCTTCGCAGTTCCCAGGGCTCTTACGCATTTGATCC*T*C
