# Supplementary material for: Non-viral in vivo electroporation-based chromosomal engineering and repair assessment in the murine uterine epithelium
Source: PLoS One. 2026 May 11;21(5):e0348797. doi: 10.1371/journal.pone.0348797 (PMC13160296; doi:10.1371/journal.pone.0348797)
Supplement: S3 Table — (PDF) [file pone.0348797.s005.pdf]

S3 Table. Primers used in the present study.

Target loci	Genomic location (mm10/GRCm38)	Sequences (5′–3′)
**Genotyping primers**		
Hmga2-1st-F	chr10: 120415727 to 120415748	CCCCAGGGAAGGTAAATAATGT
Hmga2-1st-R	chr10: 120416637 to 120416658	TTGCTCTGGACAACATTCATTC
Hmga2-2nd-F	chr10: 120416078 to 120416099	GTCTGCCATGATGTTTGTTAGC
Hmga2-2nd-R	chr10: 120416271 to 120416292	AAGTGTTGAAGAGCAGAAAGGC
Wif1-1st-F	chr10: 121049111 to 121049132	TGACCCTCCACCATTAAAATTC
Wif1-1st-R	chr10: 121049971 to 121049992	AGATCATTGCTGGGAAGAAGAG
Wif1-2nd-F	chr10: 121049423 to 121049444	GTTCAGTAGCTGGAGGAGGATG
Wif1-2nd-R	chr10: 121049610 to 121049631	GGCTTTTTATGAAAGGCAACTG
Rassf3-1st-F	chr10: 121431397 to 121431418	GAAGACAGAGACAAACCATGCC
Rassf3-1st-R	chr10: 121432300 to 121432321	CTCTTTGTGGCACTTCTGTGTC
Rassf3-2nd-F	chr10: 121431721 to 121431742	GAGATTGCTCAGGACAGAGTGA
Rassf3-2nd-R	chr10: 121431917 to 121431944	TGTACTCATACAAAATTAATCACAAGGG
Eef1a1N-1st-F	chr9: 78476997 to 78477018	ATCCATGTGGAGCCTTTATCTC
Eef1a1N-1st-R	chr9: 78477804 to 78477825	GTTAGCCTGACTTTGGCAGAAC
Eef1a1N-2nd-F	chr9: 78477215 to 78477236	GCACTTAGCTTTCACGTGTTTG
Eef1a1N-2nd-R	chr9: 78477444 to 78477465	TCAAGACCTACCTGGGAAATTG
Atf4N-1st-F	chr15: 80258094 to 80258115	CCATTGGGTTTGCTAATCTAGG
Atf4N-1st-R	chr15: 80258876 to 80258897	TGCTACTAGAGGTCTTGGGGAC
Atf4N-2nd-F	chr15: 80258497 to 80258518	TGTGGGTAGGATGATACAGCAG
Atf4N-2nd-R	chr15: 80258705 to 80258726	CCAAACCTGAGCTGGTCTATTT
Ypel4N-1st-F	chr2: 84738389 to 84738410	ATCTAATCTTTGCCATTGGGTG
Ypel4N-1st-R	chr2: 84739219 to 84739240	AACAACGCCTGGCAATTAATAC
Ypel4N-2nd-F	chr2: 84738693 to 84738714	TTGTACTGTCCTCCGTATGCAC
Ypel4N-2nd-R	chr2: 84738873 to 84738894	CCAGGGGACTGAAGACACTTAG
In(6)1J-left-1st-F	chr6: 63000428-63000449	TCCCCAAAACATCACAATACAA
In(6)1J-left-1st-R	chr6: 63001303 to 63001324	GATGGGGTTGACATTCCATAGT
In(6)1J-left-2nd-F	chr6: 63000607 to 63000628	CCCTCCAGATTTTATCCCTCTC
In(6)1J-left-2nd-R	chr6: 63001001 to 63001022	GCAGCTAAAAGAGTCAGCTTCA
In(6)1J-right-1st-F	chr6: 120826810 to 120826831	AGTTAGTCCATGTTGTGGGCTT
In(6)1J-right-1st-F	chr6: 120827732 to 120827753	ATACTCATACACAGGCACGCAC
In(6)1J-right-1st-F	chr6: 120826900 to 120826921	TCACTGCAGAAATTCTTGGAAA
In(6)1J-right-1st-F	chr6: 120827428 to 120827449	TCGTAATAAGGGCATTTCACCT
Ncoa2-1st-F	chr1: 13160923 to 13160944	CTGGGAGTTCCAAACTATCAGC
Ncoa2-1st-R	chr1: 13161602 to 13161623	GAAGTGAGTCCTGAGACAAGGG
Ncoa2-2nd-F	chr1: 13161102 to 13161123	CACACACACAGCACTTCAACAC
Ncoa2-2nd-R	chr1: 13161429 to 13161450	CACTTAGAATCCTGAGCCTTGG
Greb1-1st-F	chr12: 16733972 to 16733993	AGCCGTCACAAACTTCTCTTTC
Greb1-1st-R	chr12: 16734652 to 16734673	GGTTGCCCATCACTGTTAGAAT
Greb1-2nd-F	chr12: 16734188 to 16734209	CAGTAACATCAGCTGGCACTCT
Greb1-2nd-R	chr12: 16734562 to 16734583	ATCTGGGGAGACAGATGCTTTA
Ywhae-1st-F	chr11: 75760505 to 75760526	GTCTTGCCTGCGTATATGATTG
Ywhae-1st-R	chr11: 75761127 to 75761148	AATTCGAGACAGGGTTTCTCTG
Ywhae-2nd-F	chr11: 75760679 to 75760700	TAGCCCAAGTGTGGGTTTTATT
Ywhae-2nd-R	chr11: 75761007 to 75761028	TTCTCCTTAAGGCTGACAGAGG
Nutm2-1st-F	chr13: 50467549 to 50467570	GTGTGGAGAGAGCTCTGGACTT
Nutm2-1st-R	chr13: 50468200 to 50468221	AACTGAAACCAATAGCACTCCG
Nutm2-2nd-F	chr13: 50467737 to 50467758	TGGTCCAGAGAAGGATCTTAGG
Nutm2-2nd-R	chr13: 50468110 to 50468131	AGGAAACTGCAGAGATAGTGCC
Adamts20-1st-F	chr15: 94347286 to 94347307	AGGAGGGACATCAGGTTACAGA
Adamts20-1st-R	chr15: 94348147 to 94348168	TGTGATGACCACTGCATTATGA
Adamts20-2nd-F	chr15: 94347562 to 94347583	TCTCTGAAACTCGCAGACTGAC
Adamts20-2nd-R	chr15: 94347823 to 94347844	TTCCTGTGTGTGTGCTTCTTCT
K18N-1st-F	chr15: 102034486 to 102034507	CCTTTGTTCCGTTGATCATGTA
K18N-1st-R	chr15: 102035201 to 102035222	GAAACTGGAGGGTCACAGGTAG
K18N-2nd-F	chr15: 102034680 to 102034700	GGCATCAAATGTGTCTTCTCA
K18N-2nd-R	chr15: 102034945 to 102034966	TGATGTCTGTGGCCTTTACTGT
