# Supplementary material for: Non-viral in vivo electroporation-based chromosomal engineering and repair assessment in the murine uterine epithelium
Source: PLoS One. 2026 May 11;21(5):e0348797. doi: 10.1371/journal.pone.0348797 (PMC13160296; doi:10.1371/journal.pone.0348797)
Supplement: S4 Table — (PDF) [file pone.0348797.s006.pdf]

S4 Table. Discordant read pairs identified by whole-genome sequencing of edited uterine tissue.

Source	QNAME	RNAME	POS	RNEXT	PNEXT	CIGAR	MAPQ
Ypel4Atf4_ssODN_R3_sorted.bam t(2;15)	LH00220:459:23275YLT4:2:2387:40301:20982	chr2	82950211	chr15	80100654	150M	60
	LH00220:459:23275YLT4:2:2383:46959:14214	chr2	84231031	chr15	78732309	150M	60
	LH00220:454:22YFMJLT4:3:2415:39080:28618	chr2	85232576	chr15	80232406	150M	60
	LH00220:454:22YFMJLT4:3:1475:39970:11299	chr2	85911959	chr15	80802646	150M	60
Ncoa2Greb1_ssODN_L7_sorted.bam t(1;12)	LH00220:459:23275YLT4:3:2350:38093:7488	chr1	12008111	chr12	15093033	150M	60
	LH00220:459:23275YLT4:3:2287:6979:20996	chr1	12125600	chr12	16604952	150M	60
	LH00220:459:23275YLT4:3:2378:10206:25381	chr1	12264087	chr12	15341572	150M	41
	LH00220:459:23275YLT4:3:2445:42809:3242	chr1	12284036	chr12	18731025	150M	60
	LH00220:459:23275YLT4:3:2409:27576:15615	chr1	12740948	chr12	18335413	150M	60
	LH00220:459:23275YLT4:3:1250:18677:1743	chr1	12959657	chr12	15306529	150M	60
YwhaeNutm2_ssODN_L8_sorted.bam t(11;13)	LH00220:459:23275YLT4:3:2122:5935:25241	chr11	74658964	chr13	50055077	150M	60
	LH00851:42:22YMFJLT4:2:2491:15222:1112	chr11	76331292	chr13	51548022	150M	60
	LH00220:459:23275YLT4:3:1209:51587:11551	chr11	76394226	chr13	49680544	18S132M	60

Source: sequencing sample ID and chromosomal translocation notation; QNAME: read name; RNAME: reference sequence (chromosome) of the read; POS: leftmost mapping position; RNEXT: reference name of the mate; PNEXT: mapping position of the mate; CIGAR: alignment string describing read–reference matches; MAPQ: mapping quality score (maximum = 60).
