# Supplementary material for: Non-viral in vivo electroporation-based chromosomal engineering and repair assessment in the murine uterine epithelium
Source: PLoS One. 2026 May 11;21(5):e0348797. doi: 10.1371/journal.pone.0348797 (PMC13160296; doi:10.1371/journal.pone.0348797)
Supplement: S5 Table — (PDF) [file pone.0348797.s007.pdf]

S5 Table. Nucleotide sequences of discordant read pairs identified by whole-genome sequencing.

Source	QNAME	Read sequence (5'→3')	Mate sequence (5'→3')
**Ypel4Atf4_ssODN_R3_sorted.bam**<br>**t(2;15)**	LH00220:459:23275YLT<br>4:2:2387:40301:20982	AAAAACAGGAGTTTTAATATATCTAAAGAG<br>ACCATAAACTAAATAGCTGGATTTACTCAG<br>TATTTCCCATTCATAATCACTAATTAGCAT<br>TCCATTTATGTATCTGAAATATCCCAATTC<br>ATGAATCTGAAAGCATAATGATGTTCCACC	GTGGGGTACTTCTATTGGCTTCAGCTTCCC<br>CGGGGCTGCAGGCTGGTACAAGGTTTCATG<br>G
	LH00220:459:23275YLT<br>4:2:2383:46959:14214	GGACATGATTTTTTTTGCAGGCAGAATCCT<br>GAGGTGGATCAAAGTATGACATGGCAACAT<br>ATAGGGAGTGTGTGTGTGTGTGTGTATGTG<br>TGTGTGTGTGTGTGTGTGTGTGTGTGTGTG<br>TGTGTGTGTGTAGGAGAGACAGcGTTTcTT	TGCATCCATGAACAAGTGTATCAATATGAA<br>TGCAGGCACCACACATACACACATACA
	LH00220:454:22YFMJLT<br>4:3:2415:39080:28618	CGATATTAGAGGTGACTAAATTATAGACTC<br>ATATTGTTCTGGCTTCACATCACATCTTTG<br>TCTTTAGTACCAAAGCTATGAGCAAAGGTA<br>CAAGTCAAATCTCAGTTCACCATTTATTAA<br>AGGGAATTTTAAAAATTATTATTGCTGTTG	AGTCCCATGAGGTCATCTTCTTTGTCCTCC<br>TTCAGCACTGAATGCATCCCAGGGCAATGT<br>GTGTACTAGGCAAGTGCATTCACAGGAGCA<br>AAGCAAGGCTCAGGAAACTGAACCCGCTGC<br>CCCAAGTGTACCACCAGCACAAAGGCCCTT
	LH00220:454:22YFMJLT<br>4:3:1475:39970:11299	CACTCAGGATGATTAACAGAATTGCAGCTT<br>TTTTTCAACTGCTGAACAACTGCTTAAACT<br>GTTTTCAGCAGGAAAACAAACAATGGCAGA<br>TATGATTTTCATACTTTCTATATAATTAAT<br>TATGATGAATATTTGAAATTAGTGTGACAA	TGTGTATAGGACGAAATTCTATAATTTCCT<br>TTACTGATAATCTGGGGTTGAATAGTAATT<br>TTTTTTTCTGATAGTCCTAGAGCAGTGGAC<br>TTCGTAATGCTGCAATCCTTTAATACAGTT<br>CCTCATGTTGTGGTAACCCCCAACCACAAA
**Ncoa2Greb1_ssODN_L7_sorted.bam**<br>**t(1;12)**	LH00220:459:23275YLT<br>4:3:2350:38093:7488	AATATATTTAACAAGTAATACATGGTAGAG<br>TTCATACAAAAACCCACTGGACAGAGTAGC<br>AGACAGTTTGATGAGTGTCACCTGGCCAGC<br>AGGTATTCAACTTAGTACCAGGAACATTTT<br>GCTGTTTTATTTTGTTTATTTATTCTGAAT	ATCAAGGACTACAGGCTTCAGAATTCTCTT<br>TGGCACAGAAGATAACAAATTTTTTTAATT<br>CAGTTCTTCTGAAGATTGCAAGGCTTATAT<br>ACTGATTGAATAAGGATTATTCACAATATG<br>CAGAGAAATCTGATTTATTCAAAGTACATA
	LH00220:459:23275YLT<br>4:3:2287:6979:20996	GCCCAGCTTTAGTGGCATTTTCTATAATCA<br>AAAACCCTGCAGGATTCTGGAGCCTCTGGA<br>CATCAGAAGGTAAAAGGCAATCAGCACAGC<br>CATGACGAGGTCATTTTAACACTACCCTAA<br>AAGGTCTGGGCTCTGAGTGGGACAGGGATC	AAAGGTGGAAAGTTATTAAGGAGAAATGAA<br>TCTTCCAGGTAGTAACAGTGCCACCACACT<br>TGTGTCAGGACTCTGCCGGGGTGTCACACT<br>AGCTTCCAAGGCTTTCTCAGCATCAAGGTG<br>CACCTTGGCTCCTTCCTTTCTGTCACTGTA
	LH00220:459:23275YLT<br>4:3:2378:10206:25381	ATAAAGTTTGCAAGTCCAATGGGCCTGTCT<br>TTGCAGTGATGGCCGACTAGGCCATCTTTT<br>GATACATATGCAGCTAAAGACAAGAGCTCC<br>CGGGTACTGGTTAGTTCATATTGTTGTTCT<br>ACCTATAGGGTTGCAGTTCCCTATAGCTCC	CCGGGAATCCATCCCATAAACAACCACCAA<br>ACCGAGACACTACTGTATATACCAACAAGA<br>TTTTGCTGACAGGACCCTGATATAGCTATC<br>TCTTGTGAGGCTATGCCAGTGCCTGGCAAA<br>TACAGAAGTGGATGAAACAAAGGGTCCCCA
	LH00220:459:23275YLT<br>4:3:2445:42809:3242	GGGTTTCTATTCCTGGACAAAACCTCATGA<br>CTAAGAAGCAAGTTGGGGAGGAAAGGGTTT<br>ATTCAGCTTACACTTCCAAACTGCTGTTCA<br>TCACTAAAGGAAGTCAGGACTGGAAATCAA<br>GCAGGTCAGGAAGCAGGAGCTAATGCAGAG	TGGAGAAGAGCAAATAGAGCCATCAAGGTC<br>AGAAGGCTTTACCCTAGGTGTACTGGCTGG<br>TTTTATGTGTCAACTTGACACAGCTGGAGT<br>TATCACAGAGAAAGGAGCTTCAGTTGAGGA<br>AATGCCTCCATGAGATCCAACTGTAAGGCA
	LH00220:459:23275YLT<br>4:3:2409:27576:15615	TTAAAGGGAGAAACTCAGTGAGTCAGTGAG<br>ACTAGGCATGGGACGGCCTTGGGCCTCTTC<br>TTAAGTTAATTATGAAATCTGAGAAGAGAA<br>ACAAAAAGAACAAAAAAGATCCTCCCGCCA<br>GTTGGTGGTGCACTCCTTTAATACCAGCAC	CATGTCTTTAATCAGAGCTTATAGGAGACA<br>GGAGGCTCTCTGTGAGTTCAAGGCTAACCT<br>GGTTAAAAAAGGGAGTTCTAGGACAGCCAG<br>GGCTGTTACACAGAGAAACCCTGTTTCAAT<br>AAACAAAACAAAAGGGAGGAAAGGAGGGAG
	LH00220:459:23275YLT<br>4:3:1250:18677:1743	GTTCAGGAATTTTTCCCCTGTGCCCATATC<br>TTTGAGGCTTCCCCCCACTTTCTCCTCTAT<br>AAGTTTCAGTGTCTCTGGTTCTTGTTTGTT<br>TGCTTGCTTGTTTGTTTGTCAAGACAAGGT<br>TTCTCTGTATAGCCCTGGCTGTCATAGAAC	ACAAAAAGGCCATCAACAGgTTGGGAAAGG<br>ATcTTTACCAATCCTAAATCTGATAGGGGA<br>CTAATATCCAATATATATAAAGAACTCAAG<br>AAGGTGGACTtCAGAAAATCAAATAACCCC<br>ATTaAAAAATGGGGCTCAGAaCTgAACAAA<br>G
**YwhaeNutm2_ssODN_L8_sorted.bam**<br>**t(11;13)**	LH00220:459:23275YLT<br>4:3:2122:5935:25241	GCTCTCTGCCTGTGATCTCAGTAACCTGGG<br>GACTGAGGCAGCCATAGCACTGAATTGCAG<br>GGCAGTCTATGTTACATAACTCTAAGGTAG<br>CAGGAACTATAGAGATCTTGTCTTCTACTT<br>TCAAACAACCAACCCTCTCACCCCCATGAA	GTATTTGGTTGCAGCTTGCCTTTTCCTGTG<br>CATTTGATAGTTCTGCTCTGTGGGGG
	LH00851:42:22YMFJLT4<br>:2:2491:15222:1112	CTTGGGCAGTCTTCCCTGACTTGTTCCAAC<br>AGGCAGGTACCGGCTAGAGGtAACTGTCAC<br>GCAGTGTCTGCGGGCTATCACACCTGTGTA<br>TAGAGGAGTAGAGAGACAGGTAACACAACA<br>TTGTGCCCTTCCCTTCCACAGCCTGTTGTG	TAGACTCAAGCAAGCACAGCTTCACATAGA<br>CTCACAGAGACTAAAACACAAACATGGAGT<br>CTGCACCAGGTCCTaTGCAAAGATGTTATA<br>GCTGTCAGCTTGGTGTTCTTGTGAGACTaT<br>AATAGAAGTAGCAGGTGTATTTCTGACTC
	LH00220:459:23275YLT<br>4:3:1209:51587:11551	GGAGGCAGAGGCAGGCAGACCTCTGTTGAG<br>TTCAAGGCTAGTCTGGTCTACTGAGGGAGT<br>TCCAAGACAGCCAGGGTTACACAGAGAAAC<br>CTTGTCTTGAAAAACCAAAACCAAAACCAA<br>AAAAAAAGAGAA	CCTGGCTGTCCTGGAACTCAATTTGTAGAC<br>CAGGCTGGCCTCGAACTCAGAAATCCACCT<br>ACCTCTGCCTCCCGGGTGCTGGGATTAAAG<br>GCGTGCACTACCACGACCAGCGAAAGTTTT<br>TTAATTACCTATTTTCTAGACGAACTGTTC

Source: sequencing sample ID and chromosomal translocation notation; QNAME: read name corresponding to Table S1; Read sequence: nucleotide sequence of the read in 5' to 3' orientation; Mate sequence: nucleotide sequence of the mate read in 5' to 3' orientation. Lowercase letters indicate insertions or mismatches relative to the reference genome.
