# Supplementary material for: Non-viral in vivo electroporation-based chromosomal engineering and repair assessment in the murine uterine epithelium
Source: PLoS One. 2026 May 11;21(5):e0348797. doi: 10.1371/journal.pone.0348797 (PMC13160296; doi:10.1371/journal.pone.0348797)
Supplement: S6 Table — (PDF) [file pone.0348797.s008.pdf]

S6 Table. Summary of WGS-detected translocation-supporting read pairs and apparent junction frequencies.

Locus (breakpoints)	PE-support (discordant paired-end reads)	Properly paired reads (PPmean)	Apparent junction frequency (= PE-support / PPmean)	Percentage (%)
**Ncoa2–Greb1** (chr1–chr12)	**6**	**896,421**	**6.69×10^{-6}**	**0.000669%**
**Ypel4–Atf4** (chr15–chr2)	**4**	**825,712**	**4.84×10^{-6}**	**0.000484%**
**Ywhae–Nutm2** (chr11–chr13)	**3**	**877,046**	**3.42×10^{-6}**	**0.000342%**
