# Supplementary material for: Non-viral in vivo electroporation-based chromosomal engineering and repair assessment in the murine uterine epithelium
Source: PLoS One. 2026 May 11;21(5):e0348797. doi: 10.1371/journal.pone.0348797 (PMC13160296; doi:10.1371/journal.pone.0348797)
Supplement: S1 File — Uncropped and unadjusted gel images (related to S1A Fig). (PDF) [file pone.0348797.s009.pdf]

S1_raw_images

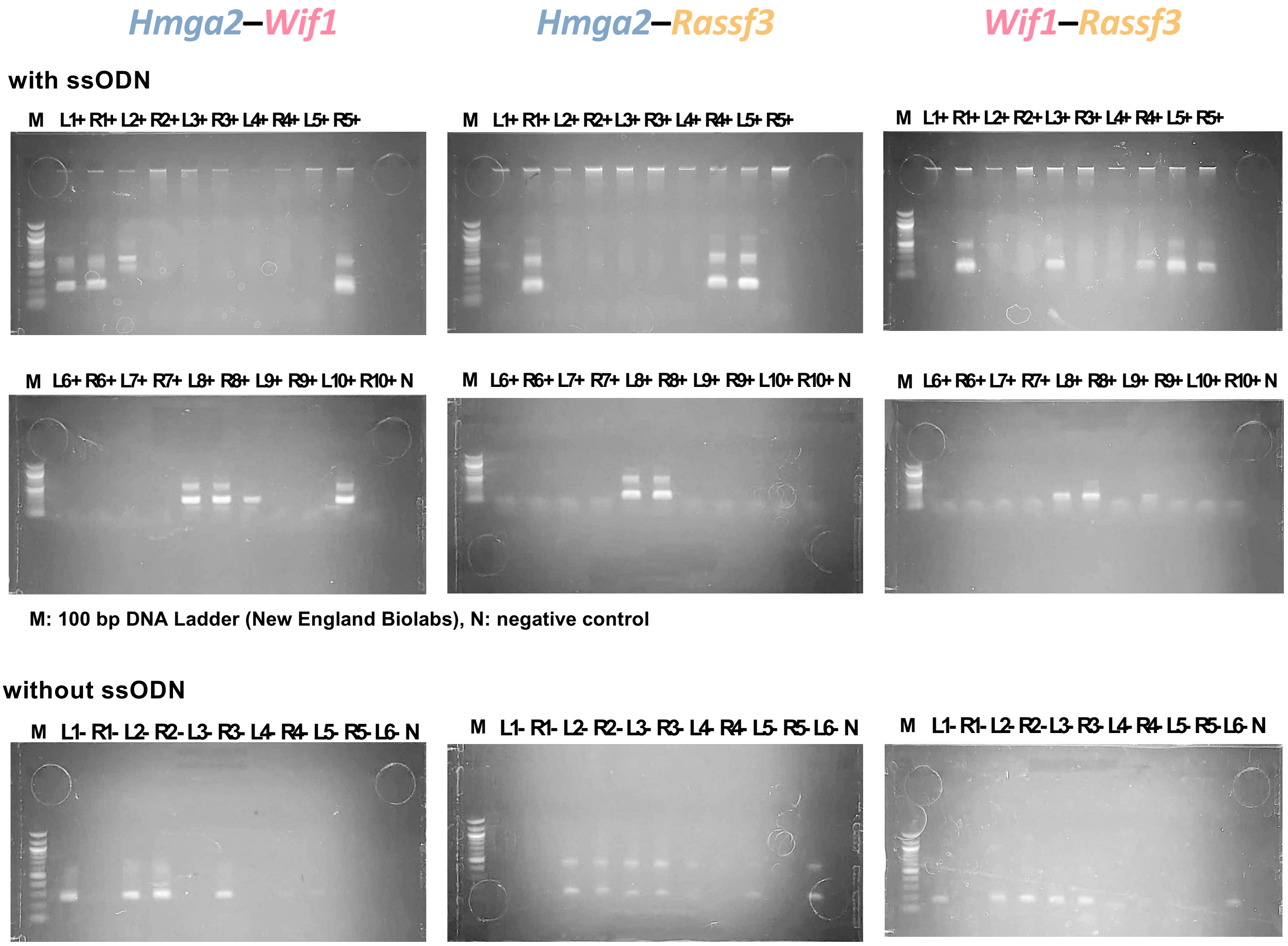

M: 100 bp DNA Ladder (New England Biolabs), N: negative control
