# Supplementary material for: Non-viral in vivo electroporation-based chromosomal engineering and repair assessment in the murine uterine epithelium
Source: PLoS One. 2026 May 11;21(5):e0348797. doi: 10.1371/journal.pone.0348797 (PMC13160296; doi:10.1371/journal.pone.0348797)

S2_raw_images

Eef1a1N–Atf4N

M #1+ #2+ #3+ #4+ #5+ #6+ N

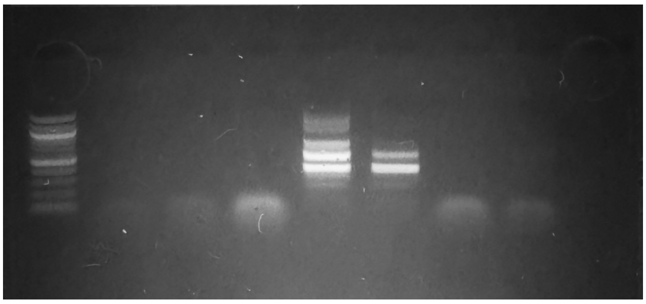

Atf4N–Eef1a1N

M #1+ #2+ #3+ #4+ #5+ #6+ N

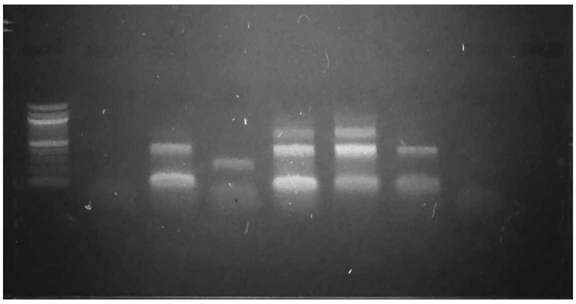

M: 100 bp DNA Ladder (New England Biolabs), N: negative control

Ypel4N–Atf4N

M L1+ R1+ L2+ R2+ L3+ R3+

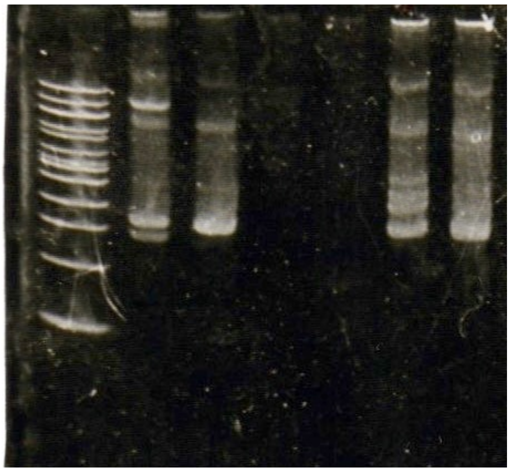

Atf4N–Ypel4N

M L1+ R1+ L2+ R2+ L3+ R3+

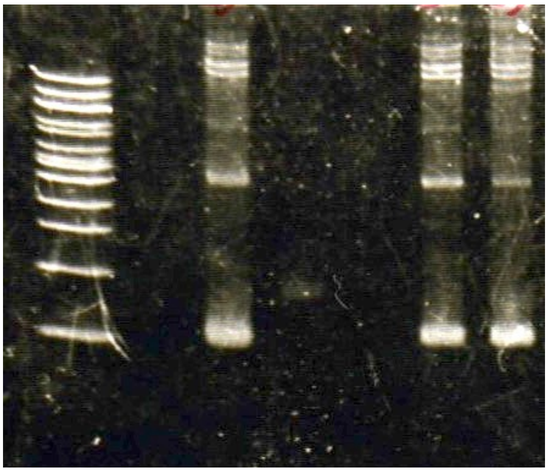

Supplement: S2 File — Uncropped and unadjusted gel images (related to S1B Fig). (PDF) [file pone.0348797.s010.pdf]
