# Supplementary material for: Non-viral in vivo electroporation-based chromosomal engineering and repair assessment in the murine uterine epithelium
Source: PLoS One. 2026 May 11;21(5):e0348797. doi: 10.1371/journal.pone.0348797 (PMC13160296; doi:10.1371/journal.pone.0348797)
Supplement: S3 File — Uncropped and unadjusted gel images (related to S1C Fig). (PDF) [file pone.0348797.s011.pdf]

S3_raw_images

In(6)1J left breakpoint | *In(6)1J* right breakpoint

with ssODN

L1+ R1+ L2+ R2+ L3+ R3+ L4+ R4+ L5+ M

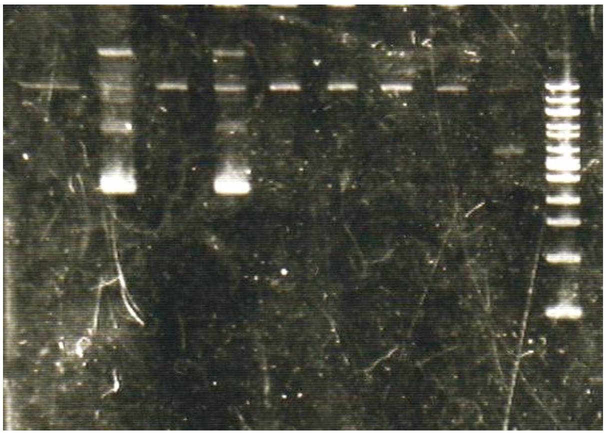

L1+ R1+ L2+ R2+ L3+ R3+ L4+ R4+ L5+ M

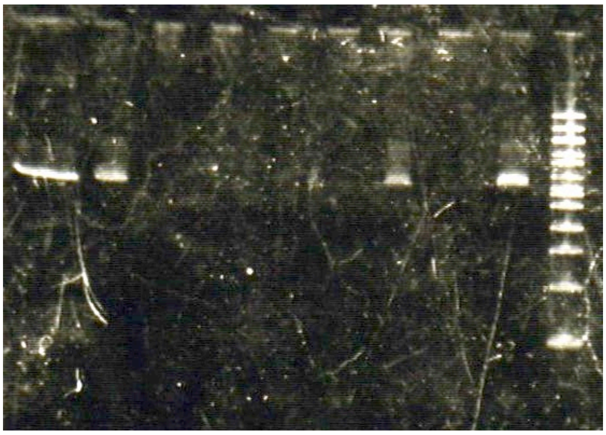

M L6+ R6+

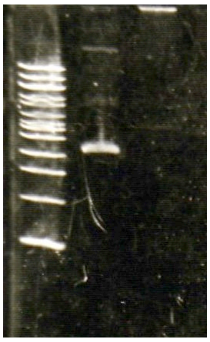

L6+ R6+

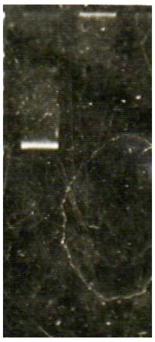

without ssODN

M L1- R1- L2- R2- L3- R3- L4- R4- L5- R5- P

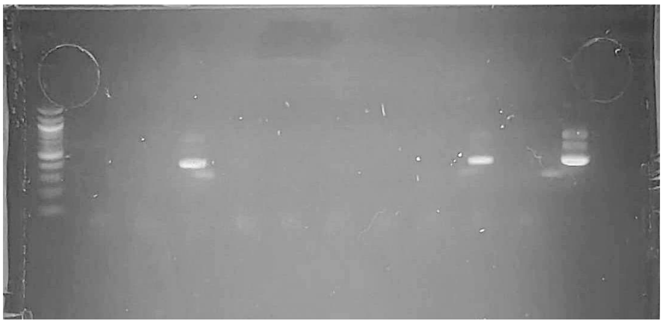

L1- R1- L2- R2- L3- R3- L4- R4- L5- R5- P

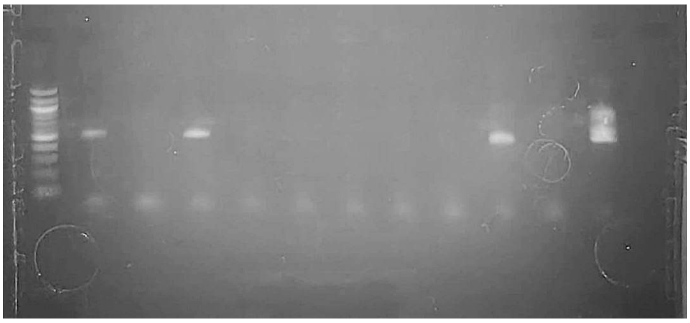

M: 100 bp DNA Ladder (New England Biolabs), P: positive control
