# Supplementary figures and images for: Non-viral in vivo electroporation-based chromosomal engineering and repair assessment in the murine uterine epithelium
Source: PLoS One. 2026 May 11;21(5):e0348797. doi: 10.1371/journal.pone.0348797 (PMC13160296; doi:10.1371/journal.pone.0348797)

*Greb1-Ncoa2* *Ncoa2-Greb1*

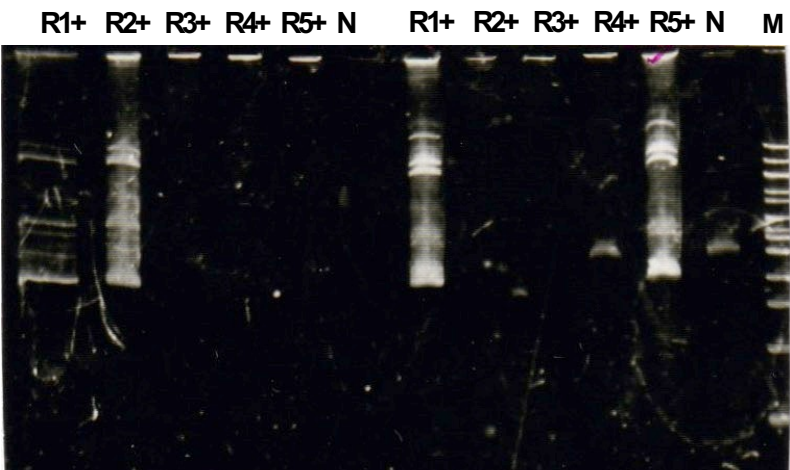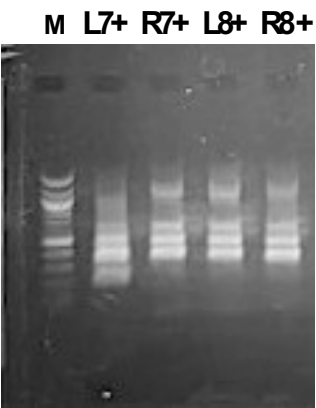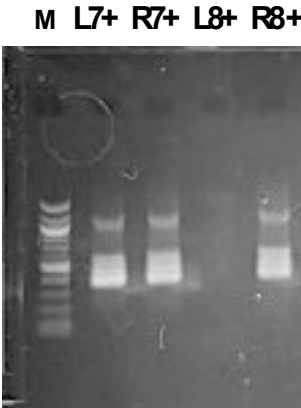

M: 100 bp DNA Ladder (New England Biolabs), N: negative control

*Ywhae-Nutm2* *Nutm2-Ywhae*

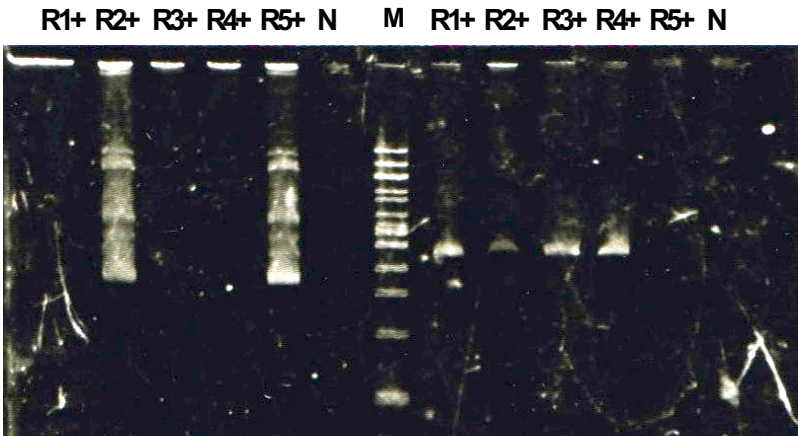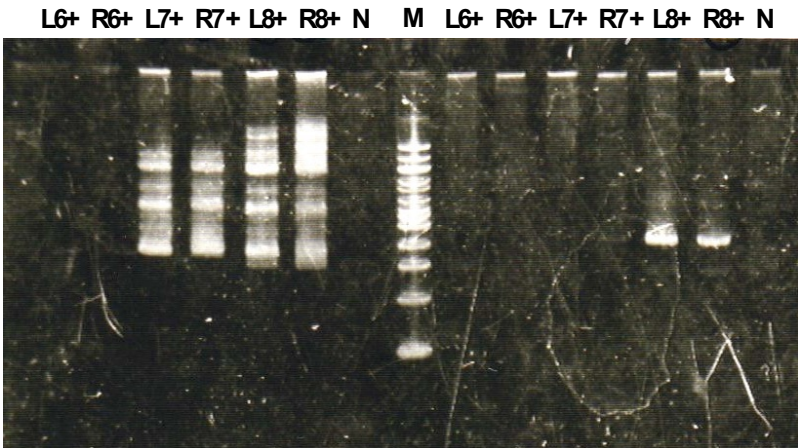

Supplement: S4 File — Uncropped and unadjusted gel images (related to S1D Fig). (PDF) [file pone.0348797.s012.pdf]
