# Supplementary material for: Non-viral in vivo electroporation-based chromosomal engineering and repair assessment in the murine uterine epithelium
Source: PLoS One. 2026 May 11;21(5):e0348797. doi: 10.1371/journal.pone.0348797 (PMC13160296; doi:10.1371/journal.pone.0348797)
Supplement: Attachment — Submitted filename: 155649_1_rebuttal_3637226_t87nv8.docx [file pone.0348797.s013.docx]

**Manuscript number:** RC-2025-02936R

**Corresponding author(s):** Satoru Iwata

## 1. General Statements [optional]

We aim to thank the reviewers for their constructive feedback, which has significantly strengthened the manuscript. The principal concern across the reviews was the absence of quantitative information on the frequency of engineered chromosomal rearrangements. To address this, we incorporated a WGS-based estimation of allele abundance, which provided molecular confirmation of the intended rearrangements while transparently reflecting the signal dilution inherent to our bulk tissue analysis.

In response to the feedback, we have carefully reframed the study as a "proof-of-concept" for non-viral somatic chromosomal engineering. We have removed subjective descriptors such as “efficient,” “novel,” and “cost-effective” to strictly align our conclusions with the quantitative data. Instead, we now emphasise the simplicity of the approach and its utility as a model system for assessing DNA repair mechanisms in a physiological context.

Specific revisions to improve clarity and transparency include:

1. **Quantitative assessment:** We added WGS analysis to estimate the apparent allele abundance and explicitly discussed the methodological limitations regarding detection sensitivity in the Results and Methods.
2. **Mechanistic insight:** We expanded the evaluation of repair signatures, specifically clarifying how FoSTeS/MMBIR-related outcomes were assessed, to highlight the system's value in distinguishing repair pathways.
3. **Methodological clarity:** We explained the rationale for whole-organ DNA extraction, refined the description of dextran localisation (Fig. 1), and clarified the distinction between *in vitro* and *in vivo* prime-editing outcomes.

These changes ensure that the manuscript accurately presents the method as a feasible, chemically defined strategy for exploring chromosomal biology, acknowledging the current efficiency constraints while highlighting its potential for mechanistic studies.

## 2. Description of the planned revisions

All planned revisions have been completed and incorporated in the revised manuscript. Details are provided in Section 3.

## 3. Description of the revisions that have already been incorporated in the transferred manuscript

### **Reviewer #1**

**Major Comment:**
*The authors show that chromosomal aberrations occur, but they provide no relative efficiency or quantification. To demonstrate a method, this is a fundamental problem. The method could work at 0.0000001%, 1%, or 10% of cells/alleles—and this difference is crucial. A manuscript of this nature should not only show that something works sometimes, but also how well it works, in order to establish a baseline for future adoption and improvement of the technique.*

*Quantitative PCR could provide an estimation of the efficiency, or the whole-genome sequencing (WGS) data could be further analyzed to get such estimations.*

*Without this quantification, it is difficult to justify the authors' claims that their method is "efficient". Furthermore, other strong adjectives such as "novel" or "cost-effective" should be moderated or avoided, as these are debatable claims. Avoiding overclaims will not impair the manuscript's overall impact.*

**Response and manuscript revision:**

We thank the reviewer for this crucial insight. We have now incorporated a WGS-based estimation of allele abundance to establish a quantitative baseline.

Specifically, for each targeted locus, we calculated the ratio of discordant paired-end reads to properly paired reads within a 2 Mb window. As genomic DNA was extracted from the entire uterine wall, these values reflect the signal dilution inherent to the procedure. We have explicitly clarified this limitation in the Methods and Results section.

The estimated abundance was consistently low—approximately 3.4×10⁻⁶ to 6.7×10⁻⁶ (0.00034–0.00067%). However, the consistency of these values across independent loci demonstrates that the method is robust enough to serve as a tractable evaluation system for studying repair outcomes, despite the low efficiency.

Accordingly, we have reframed the study as a “proof-of-concept” for a simple, non-viral approach. We removed descriptors such as “efficient,” “novel,” and “cost-effective” to strictly align with the data, focusing instead on the method’s validity for assessing chromosomal mechanisms in a physiological context.

Below, we list the revisions made to the text:

- **Methods section (lines 250–255):**

“To obtain a minimal quantitative estimate of junction-supporting reads in bulk uterine tissue, we calculated the apparent editing frequency at each locus by dividing the number of discordant paired-end reads (PE-support) with the number of properly paired reads within the corresponding ±2 Mb window (PPmean). This simple ratio (PE-support/PPmean) reflects the relative abundance of rearranged alleles in the sequenced tissue, without assuming a clonal structure or purity.”

- **Results section (lines 482–486):**

“We further estimated the relative abundance of rearranged alleles in the bulk DNA library by comparing discordant paired-end reads to properly paired reads. The apparent abundance was consistently low, ranging approximately from 3.4×10⁻⁶ to 6.7×10⁻⁶ (0.00034–0.00067%; Table S2). This frequency is consistent with the expectations of somatic mosaicism combined with whole-organ tissue dilution.”

**Minor Comment 1:**
*I do not see how DNA extraction from the whole organ removes bias if only the epithelium is specifically targeted. This point requires clarification.*

**Revised Response:**

We appreciate the reviewer's careful observation. The reviewer highlights an apparent contradiction: if *in vivo* electroporation specifically targets the epithelium, why extract DNA from the entire uterine horn including the stroma and myometrium?

We selected the whole-organ approach after quantitatively evaluating the trade-offs between "spatial selection bias" and "signal dilution."

1**. The Rationale:** Quantifiable Dilution is Better than Unpredictable Bias

- **Risk of Epithelial-Only Sampling:** *In vivo* electroporation creates mosaic editing patterns. Some regions are "hot spots" (high efficiency due to electrode contact) while others are "cold spots." Sampling a small epithelial fragment introduces unpredictable spatial bias—potentially overestimating efficiency by 10-100x if a hot spot is selected, or missing events entirely in a cold spot.
- **Benefit of Whole-Organ Sampling:** By extracting DNA from the entire uterine horn (~8-10 mm), we generate an unbiased spatial average. Although this introduces "signal dilution" (since the epithelium comprises only ~5% of the tissue mass), this dilution is a constant, calculable factor.

2. **Why This is Scientifically Superior for Robustness:**

It is scientifically more rigorous to measure a "diluted but representative average" than to rely on "highly specific but spatially random" samples. This ensures that our WGS quantification reflects the method's true, reproducible performance across independent animals, rather than local variations. We can mathematically account for the dilution (observed frequency ÷ epithelial fraction) to estimate true cellular efficiency.

We revised the manuscript accordingly.

- **Methods section (lines 199–204):**

“This comprehensive whole-organ approach was selected to mitigate potential sampling bias that may arise due to the region-dependent variability of *in vivo* electroporation. By avoiding the collection of solely highly or poorly edited regions, we aim to ensure that the observed outcomes accurately reflect the spatially averaged effects, accepting the trade-off of signal dilution by non-target stromal tissues.”

**Minor Comment 2:**
*The reproducibility of the method cannot be fully assessed without the quantification of efficiency. As presented, the authors show that it works at different loci; however, its reproducibility on a single locus remains unclear. It is paradoxical that the authors, in the introduction, claim there is no reproducible method to generate such anomalies, yet they do not sufficiently demonstrate the reproducibility of their own method either.*

**Revised Response:**

We thank the reviewer for highlighting this inconsistency. We agree that our original framing created a paradox. To resolve this, we have substantially revised the Introduction:

1. **Removed criticisms of viral methods:** We deleted the argument that existing viral methods are limited by "effort, cost, or safety risks."
2. **Removed claims of efficiency:** We explicitly deleted the claim that our method is an "efficient" alternative.
3. **Reframed the study:** We now position the study as a proof-of-concept for a chemically defined, simple platform designed to evaluate chromosomal repair mechanisms.

This realigns our objective from "replacing viral vectors" to "providing a specific tool for mechanistic analysis." Additionally, regarding reproducibility, we clarified in the Results that the intended rearrangements were consistently detected in multiple independent animals, confirming that the method serves as a reproducible model system despite the lower efficiency.

**Minor Comment 3:**
*It is difficult to assess whether the dextran is truly inside the epithelial cell, or just at the surface, based on the provided image. Separated channel images and specific details on the image acquisition (e.g., section thickness relative to cell size, and image section size) would be highly helpful. If the section is as large as the cells, it is difficult to confidently conclude that the signal is intracellular rather than surface-bound.*

**Revised Response:**

We appreciate the reviewer’s careful observation. We agree that because the paraffin sections were cut at a thickness of 5–7 μm—similar to the diameter of individual epithelial cells—the fluorescence signals cannot be unambiguously resolved as strictly intracellular versus surface-bound. Our primary objective with this image was to demonstrate the spatial restriction of the delivery to the luminal epithelial layer, contrasting it with the underlying stroma and myometrium, rather than to claim definitive cytoplasmic localisation.

To improve data transparency, we have updated Figure 1B to include separate Dextran-TMR and DAPI channels, enabling readers to independently assess the signal distribution relative to the epithelial nuclei.

**Minor Comment 4:**
*It is a bit confusing when the authors state that PETI allows efficient prime editing chromosomal rearrangement in human cultured cells, but that there is no chromosomal rearrangement induction using prime editing in vivo. I assume the authors refer to the mouse model and non-cultured cells. Please clarify this distinction.*

**Revised Response:**

We thank the reviewer for pointing out this ambiguity. Our intention was to contrast the established success of prime editing for rearrangements in *in vitro* human cell culture models (e.g., PETI) with the current lack of reported success in *in vivo* somatic tissues.

To clarify this distinction, we have revised the Introduction to explicitly state that while PETI exhibits high precision in cultured cells, the induction of defined chromosomal rearrangements directly in somatic tissues *in vivo* remains technically challenging.

We revised the manuscript accordingly.

- **Introduction section (lines 52–57):**

“Recently, advanced prime editing techniques, such as prime editing with wild-type Cas9 (WT-PE) and prime editor nuclease-mediated translocation and inversion (PETI), have exhibited high precision in inducing rearrangements in cultured human cells [12], [13]. However, despite these *in vitro* advances, the induction of defined chromosomal rearrangements directly in somatic tissues *in vivo* remains technically challenging.”

**Minor Comment 5:**
*An explanation of how the authors checked for FoSTeS/MMBIR and how they arrived at their conclusion about these mechanisms would be useful.*

**Revised Response:**

Thank you for highlighting this point. We evaluated the repair junctions for specific structural signatures characteristic of FoSTeS/MMBIR, such as localised duplications, triplications, or extended microhomology, which we previously defined in Recql5-mutated embryos. In the present study, the junctions validated by PCR and Sanger sequencing—and further inspected by WGS—did not show any of these complex features; instead, they exhibited either precise repair or simple indels consistent with canonical NHEJ/MMEJ. Therefore, while we acknowledge the limits of bulk detection for extremely rare events, our statement has been revised to report this positive finding: the recovered junctions suggest that somatic repair in the uterine epithelium via this method is predominantly free of the complex structural errors associated with FoSTeS/MMBIR.

We revised the manuscript accordingly.

- **Results section (lines 429–434):**

“Most notably, sequence analysis revealed that the junctions were free of the complex signatures typically associated with FoSTeS/MMBIR, such as extended microhomology or localized copy number gains. This indicates that, unlike the chaotic repair observed in *Recql5*-mutated embryos, repair outcomes in somatic uterine epithelial cells via this method are predominantly defined by simple ligation events without complex structural errors.”

**Minor Comment 6:**
*Perhaps Table 1 could be moved to the Supplementary Data.*

**Revised Response:**

Thank you for this helpful suggestion. Table 1 has been moved to Supplementary Table S1, and all in-text citations have been updated accordingly.

### **Reviewer #2**

***The manuscript describes an electroporation method for direct manipulation of uterine epithelium of the mouse using CRISPR/Cas9 nucleases and bridging ssODNs. The generation of chromosome deletions, translocations and inversions is demonstrated in pooled tissue by simple PCR. Despite the positive results by PCR, the manuscript lacks a depth of analysis or the verification of the genomic manipulations using an independent technique and fails to provide any quantification of the extent of extent of the genetic modification within the electroporated tissue i.e. the efficiency.***

***These shortcomings were all identified by many independent reviews during the Review Commons assessment of the paper and the authors have now submitted an extensive revision of the manuscript.***

***In my opinion, the revision merely tightens up the technical details of the experiments but fails to add the extra information required by the review to fully demonstrate the utility of the approach.***

**General Response:**

We thank the reviewer for their critical assessment. We take the reviewer’s point regarding the need to demonstrate the utility of the approach beyond mere technical description.

To address the concerns regarding verification and depth, we have incorporated Whole-Genome Sequencing (WGS) analysis. This addition provides:

1. **Independent Verification:** Molecular confirmation of the structural rearrangements (translocations and inversions) by a technique orthogonal to PCR.
2. **Quantification:** An estimation of allele abundance, providing the quantitative baseline requested.

Crucially, we have reframed the study to clarify its specific utility. Rather than proposing a high-efficiency gene therapy tool, we present this method as a simple, chemically defined platform to evaluate the mechanisms of long-range chromosomal joining (e.g., interaction between distant genomic loci) in a somatic context. Unlike viral methods, this "hit-and-run" RNP approach allows for the observation of structural variation formation (such as FoSTeS/MMBIR signatures) without the confounding effects of viral integration or prolonged nuclease activity.

We believe this clearer focus on the mechanistic evaluation of structural variations, supported by the new WGS data, fully demonstrates the scientific value of the approach as a proof-of-concept.

**Major comment 1:**
*Many of the reviews require the authors to provide cytogenetic confirmation of the chromosome manipulations. The authors claims that this would be technically challenging and beyond the scope of the revision. This is a weak argument as there are a number of approaches that could be tried. For example, the use of a fluorescently tagged Cas9 protein could be used to perform FACs enrichment of the edited cells, followed by interphase fiber-FISH. Following suggestions from the reviewers, the authors do monitor the spatial delivery of the gene editing reagents using TMR co-injections, whish is helpful, but this is simply used for histology and is not used to help isolate the editing cells.*

**Response and manuscript revision:**

We appreciate the reviewer’s suggestion regarding cytogenetic validation. We seriously considered performing FACS enrichment followed by fiber-FISH. However, we prioritized Whole-Genome Sequencing (WGS) because it aligns more directly with the specific aim of this study: to evaluate the molecular mechanisms of long-range chromosomal joining (e.g., translocations and inversions).

We argue that WGS provides a more rigorous validation for this specific purpose than cytogenetics for two reasons:

1. **Mechanistic Resolution:** The core value of our platform is to assess *how* distant genomic ends are joined in a somatic context (e.g., determining if the repair involves simple ligation, microhomology, or complex signatures like FoSTeS/MMBIR). Fiber-FISH visualizes the chromosomal structure but cannot reveal the sequence fidelity at the junction. Only nucleotide-resolution data from WGS can definitively characterize these repair signatures.
2. **Validation of Methodological Simplicity:** A key advantage of our approach is its simplicity—allowing for the induction and detection of rearrangements directly in tissue without the need for complex manipulations like single-cell dissociation or sorting. Demonstrating that these events are detectable by WGS in bulk tissue (without FACS enrichment) serves as a stronger proof-of-concept for the method's robustness and accessibility as an *in vivo* evaluation system.

Therefore, we reasoned that providing unambiguous molecular evidence of the rearrangement junctions via WGS offers higher scientific value for assessing repair mechanisms than visual confirmation. We have added a discussion acknowledging the potential of FACS-based cytogenetics for future applications requiring cell-level quantification.

We revised the manuscript as follows:

- **Discussion section (lines 543–550):**
  “We also recognize the limitations regarding cytogenetic visualization. Although FISH and karyotyping are standard for visualizing rearrangements, the low proliferative index of the uterine epithelium and the rarity of edited cells made obtaining informative metaphase spreads impractical in this study. Consequently, we prioritized WGS and PCR, which provide the nucleotide-level resolution necessary to evaluate repair fidelity—information not accessible via standard cytogenetics. Future improvements in editing efficiency or enrichment strategies, such as the use of fluorescently tagged Cas9 for FACS-based isolation, will be necessary to enable direct cell-by-cell assessment.”

**Major comment 2:**
*The authors address the criticism of not providing direct evidence for editing by performing WGS using short-read sequencing. They conclude that structural variant callers were not able to detect the chromosome arrangements due to the small fraction of edited cells, and demonstration was only possible by manual curation of the data. Again - enrichment of the edited cells using a fluorescent Cas9 approach could have been used to more convincingly demonstrate the reported edited. In addition short-read sequencing approaches are an odd choice for chromosome aberrations - I would have expected long-read sequencing approaches to be more appropriate here.*

**Response and manuscript revision:**

We appreciate the reviewer’s suggestion regarding long-read sequencing. We agree that long-read technologies are generally superior for resolving the full structure of complex variants. However, we selected high-depth short-read sequencing (Illumina NovaSeq, ~90 Gb per sample) for the following practical and scientific reasons:

1. **Sensitivity for rare somatic events (Primary Reason):** As established in our Results, the edited alleles are extremely rare (approx. 10^-6^) due to the mosaic nature of the editing and tissue dilution. Detecting such low-frequency events requires ultra-deep sequencing coverage. Achieving the necessary read depth to confidently detect these rare alleles using long-read technologies remains cost-prohibitive and technically challenging compared to short-read sequencing.
2. **Validation of Junctional Fidelity:** While Sanger sequencing provided initial confirmation, we used high-quality short reads to independently validate the sequence fidelity at the breakpoints. Short reads allowed us to confirm, with high base-calling confidence, that the junctions contained the specific ligation products or small indels identified by Sanger, and were locally free of the microhomology signatures associated with FoSTeS/MMBIR, reinforcing our conclusion that the repair was predominantly precise at the junctional level.

We have clarified in the Results section that manual inspection was necessary not because the data was ambiguous, but because standard SV callers are often tuned to filter out such low-frequency somatic mosaicism as noise. The high MAPQ scores of our manually curated reads confirm their validity.

We revised the manuscript as follows:

- **Discussion section (lines 521–526):**
  “To detect and characterize these rare events, we strategically employed high-depth short-read whole-genome sequencing. Although long-read sequencing is advantageous for structural phasing, we selected short-read sequencing to ensure the ultra-deep coverage required for identifying rare somatic alleles (10^-6^) and to provide the nucleotide-level resolution necessary for validating junctional fidelity.”

**Major comment 3:**
*Even without purification of the edited cells, some degree of quantification of the editing would be helpful to add more validity to the approach. One could envisage some kind of quantitative PCR approach (Q-PCR/dd-PCR allele counting) to demonstrate a loss of signal.*

**Response and manuscript revision:**
We agree that quantification is essential. In the revised manuscript, we have addressed this by utilizing the WGS data to provide a quantitative estimate of "apparent allele abundance." We calculated the ratio of discordant paired-end reads (PE-support) to properly paired reads within the target window. This yields a reproducible quantitative value (ranging from 3.4×10⁻⁶–6.7×10⁻⁶) for each locus. While standard qPCR is difficult to normalise without a clonal reference standard for the de novo created junction, our WGS-based counting approach effectively serves as a digital quantification of the rearrangement frequency within the library. We believe this meets the reviewer’s request for validity.

**Major comment 4:**
*The reviewers question the fundamental premise of the experimental approach as being simpler than viral delivery of editing reagents. The authors revisions aimed to address this point are weak - their set-up requires extensive surgical procedures, microinjection of reagents and in vivo electroporation using a very specific equipment, not widely available.*

**Response and manuscript revision:**

We appreciate the reviewer's critical point regarding the practical complexity of our *in vivo* surgical approach. The reviewer raises an important challenge: Is our method truly "simpler" than viral delivery when it requires microsurgery and specialized electroporation equipment?

We address this concern by differentiating between "operational simplicity" and "compositional/scientific simplicity," and explicitly discussing the trade-offs:

**1. Operational Reality—Acknowledging the Reviewer's Point:** The reviewer is absolutely right: microsurgical access and *in vivo* electroporation are technically demanding and require specialized training. For researchers seeking a high-throughput, user-friendly method for mere gene delivery, viral vectors remain operationally superior.

**2. Scientific Simplicity—A Distinct Advantage:** However, the value of our approach lies not in *operational simplicity* (where viral vectors excel) but in *chemical and molecular simplicity*, which is critical for mechanistic studies:

- **Reagent Preparation:** Unlike viral vectors which require complex cloning, packaging, and titration, our method uses purified RNP and ssODN ("mix-and-go").
- **Chemical Definition:** The system is chemically defined, avoiding the complexity of viral capsids or backbone sequences.
- **Temporal Precision:** The "hit-and-run" nature of RNPs (minutes-to-hours activity) is far simpler than the constitutive expression (months-to-years) of viral vectors.

**3. Mechanistic Research—When the Trade-off is Justified:** For our specific aim—evaluating chromosomal repair mechanisms—the operational cost of surgery is justified by the scientific clarity it provides:

- **Clean Repair Analysis:** Because the nuclease activity is transient, we capture the "molecular footprint" of the primary repair event without the noise of recurrent cleavage or off-target cutting associated with prolonged viral expression.
- **No Integration:** Avoiding viral integration removes a major confounding factor in genomic analysis.

**Conclusion:** We do not propose our method as a general replacement for viral vectors. Instead, we position it as a specialized tool where mechanistic clarity and a "clean" genomic background are prioritized over operational ease.

We revised the manuscript as follows:

- **Discussion section (lines 514–521):**
  “We acknowledge that our current method yields significantly lower editing frequencies than viral vectors and requires microsurgical access. However, unlike viral methods that introduce integration/cargo complexities, our RNP approach offers "compositional simplicity" and a transient editing window. Therefore, rather than proposing this as a high-efficiency clinical alternative, we present it as a simplified experimental strategy for specific contexts—such as mechanistic analysis of repair outcomes—where a "clean" genomic background without viral integration is prioritized.”

**Major comment 5:**
*The authors fail to address the criticism of uncertainty surrounding the allelic status of the chromosomal engineering. I appreciate this is hard to achieve - especially since their analysis is based on whole sampling of the tissue post electroporation, but with some kind of single cell isolation of edited cells, this resolution would be possible.*

**Response and manuscript revision:**

We appreciate the reviewer’s comment regarding the determination of allelic status. We acknowledge that single-cell isolation would be required to definitively prove mono-allelic versus bi-allelic editing.

However, based on the mechanism of our "hit-and-run" system and the quantitative data obtained, we can scientifically infer the allelic status without single-cell sorting:

1. **Probabilistic Rarity of Bi-allelic Events:** In this somatic model, we are inducing translocations between unlinked chromosomes. The probability of two independent translocation events occurring simultaneously on both alleles in the same cell is statistically vanishingly rare compared to mono-allelic events. Therefore, the edited cells are almost certainly heterozygous, which accurately models the "first hit" scenario in somatic tumorigenesis.
2. **Quantitative Evidence from WGS:** As established by our WGS data, the variant allele frequency is approximately 10^-6^. This extremely low abundance reflects a mosaic population where edited cells are sparse. In such a non-selected context, the likelihood of bi-allelic modification is negligible.

Strategic Focus of the Study:

Our primary goal in this proof-of-concept is to provide a simple platform to evaluate the molecular mechanisms of joining (e.g., sequence fidelity at the junction). For this specific aim, knowing the precise zygosity of the cell is secondary to characterizing the DNA repair signatures (NHEJ vs. MMBIR) at the breakpoint, which we have successfully achieved via high-depth WGS and PCR.

We have revised the Discussion to clarify that, given the probabilistic nature of somatic editing, the generated rearrangements represent heterozygous events, serving as a model for early-stage somatic structural variations.

We revised the manuscript as follows:

- **Discussion section (lines 529–532):**
  “Given this rarity and the non-selected nature of the events, these rearrangements likely represent mono-allelic (heterozygous) modifications, effectively modeling the stochastic "first hit" scenarios often seen in early somatic tumorigenesis.”

**Major comment 6:**
*What would significantly improve the manuscript is some phenotypic consequence of the chromosomal manipulation. If a targeted translocation break point or chromosomal deletion results in some kind of dominant phenotype (most probably tumorigenesis), then this could be characterised and would provide a convincing demonstration of the utility of this approach (and potentially a larger source of edited cells). The authors claim that phenotypic assessment would be the subject of a follow-on study, but given the very rudimentary evidence (PCR) provided for the chromosome engineering in the manuscript, I think supplementing the manuscript with a functional exemplar would be essential before publication can be considered.*

**Response and manuscript revision:**

We strongly agree with the reviewer that demonstrating a phenotypic consequence, such as tumorigenesis, is crucial for validating this system as a comprehensive disease model.

However, we respectfully propose that for the specific goal of this *proof-of-concept study*—evaluating the immediate molecular mechanisms of chromosomal joining in somatic tissues—molecular validation via WGS provides higher scientific value than phenotypic observation.

**Why tumorigenesis is not the appropriate primary endpoint for this study:**

1. **Signal Obscuration through Clonal Evolution:** Tumorigenesis involves extended latency and secondary clonal selection. During this process, the specific signatures of the original repair event (e.g., precise ligation vs. MMBIR) can be obscured by acquired mutations and clonal dynamics. This makes it difficult to definitively distinguish the primary repair outcome from secondary evolutionary changes.
2. **Mechanistic Clarity:** By contrast, immediate molecular characterization via WGS captures the primary repair outcome in a "clean" state, before secondary modifications accumulate. This provides unambiguous insight into how somatic cells repair large-scale chromosomal breaks, which aligns directly with our study's aim.
3. **Distinct Scientific Questions:** This manuscript presents a proof-of-concept for a *methodology* to study chromosomal repair. The appropriate validation for this specific aim is molecular (does the method work technically?), not phenotypic (does it cause tumors?). These are distinct scientific questions suited for distinct studies.

**Tumorigenesis as a Logical Future Direction:** We fully recognize the scientific importance of phenotypic validation. Once the mechanistic foundation is established through this study, tumorigenesis modeling will be a logical and valuable next step. However, pursuing tumorigenesis in the current manuscript would dilute the focus on mechanistic analysis.

**Conclusion:** The rigorous molecular characterization we provide via WGS constitutes sufficient technical validation for this proof-of-concept study, demonstrating that the platform is suitable for analyzing chromosomal repair mechanisms. This work establishes the necessary scientific foundation for future studies involving phenotypic endpoints.

**Minor comment:**
*The discussion on whether providing CRISPR/Cas9 reagents as RNP leads to only transient delivery which then protects the genome from repeated cleavage is inaccurate. It is well established that, even with RNP delivery, targeted modifications that fail to silence the spacer or the PAM result is significant levels of indels.*

**Response and manuscript revision:**

We thank the reviewer for highlighting this in the revised manuscript. We agree that the delivery of CRISPR–Cas9 as an RNP does not prevent re-ligation if the repaired junction retains a functional spacer–PAM sequence. Our original wording implied full protection from repeated cleavages, which was not our intention.

In the revised manuscript, we have clarified that although mismatches present within the protospacer substantially reduce Cas9 activity at the repaired junction (Fu et al., 2013; Hsu et al., 2013), transient RNP delivery only limits the time window during which recutting could occur and does not eliminate this possibility. We revised the relevant text accordingly.

We revised the manuscript as follows:

- **Results section (lines 374–381):**
  “At the left breakpoint, the repair ssODN template was based on the wild-type sequence but also contained the protospacer adjacent motif (PAM) and upstream nucleotides matching the Cas9 target site in the *In(6)1J* genome. Due to mismatches introduced in the protospacer sequence upon repair, Cas9 activity at the restored site is expected to be substantially reduced, thereby minimizing the risk of re-cleavage [32], [33]. Although RNPs degrade relatively quickly, transient delivery further mitigates the risk of re-cutting by limiting the duration during which additional cleavage can occur.”

## 4. Description of analyses that authors prefer not to carry out

We appreciate the reviewer’s constructive suggestions. While we seriously considered these additional analyses, we strategically prioritized approaches that align more directly with the specific aim of this study: providing a simple platform to evaluate the molecular mechanisms of chromosomal joining.

- **Single-cell–level validation (FISH or karyotyping):** We prioritized Whole-Genome Sequencing (WGS) over cytogenetic visualization (FISH/karyotyping) for scientific reasons. The core value of our platform is to assess *how* distant genomic ends are joined (e.g., distinguishing precise ligation from microhomology-mediated repair like FoSTeS/MMBIR). While FISH provides visual confirmation, it lacks the nucleotide-level resolution required to verify this junctional fidelity. Therefore, we focused on high-depth WGS to provide unambiguous molecular evidence of the repair signatures, which offers higher mechanistic insight than low-resolution structural visualization.
- **FACS-based enrichment of edited cells:** We acknowledge that FACS enrichment would increase sensitivity. However, a key advantage of our proposed method is its operational simplicity—enabling the assessment of chromosomal rearrangements directly in tissue without complex downstream manipulations. Establishing a dissociation protocol carries the risk of inducing cellular stress or selection bias. By demonstrating that these rearrangements are detectable in bulk tissue via high-depth WGS, we provide a stronger proof-of-concept for the method's robustness and accessibility as an *in vivo* evaluation system. We have, however, added a discussion explicitly identifying FACS as a necessary future step for cell-level quantification.
- ***In vivo* phenotypic validation (e.g., tumorigenesis):** We focused on immediate molecular validation rather than long-term phenotypic observation. While tumorigenesis is a valuable endpoint for disease modeling, it requires long latency periods and involves secondary evolutionary events (clonal selection) that can obscure the signatures of the original chromosomal repair. To evaluate the primary repair outcomes of the "hit-and-run" editing, immediate molecular characterization via WGS and PCR provides a more accurate and "cleaner" assessment of the joining mechanisms than retrospective analysis of developed tumors.
